# Galectin‐4: A Novel Mediator of Human Intervertebral Disc Degeneration

**DOI:** 10.1002/jsp2.70194

**Published:** 2026-08-06

**Authors:** Christine Strauss, Daniel Djojic, Johannes Stadlmann, Josef Georg Grohs, Jürgen Alphonsus, Sebastian Schmidt, Sabine André, Katharina Margareta Pichler, Sara Savic‐Ivanovic, Melanie Cezanne, Mario Rothbauer, Reinhard Windhager, Stefan Toegel

**Affiliations:** ^1^ Karl Chiari Lab for Orthopaedic Biology, Department of Orthopedics and Trauma Surgery Medical University of Vienna Vienna Austria; ^2^ Ludwig Boltzmann Institute for Arthritis and Rehabilitation Vienna Austria; ^3^ University of Natural Resources and Life Sciences Vienna Austria; ^4^ Division of Orthopedics, Department of Orthopedics and Trauma Surgery Medical University of Vienna Vienna Austria; ^5^ Chair of Biochemistry and Chemistry, Faculty of Veterinary Medicine Ludwig‐Maximilians‐Universität München Munich Germany

## Abstract

**Background:**

Intervertebral disc (IVD) degeneration is associated with severe clinical symptoms including chronic back pain. Galectins are a family of carbohydrate‐binding proteins, some of which can induce functional disease markers in IVD cells and other musculoskeletal tissues. Galectin‐4 and ‐8 were shown to trigger disease‐promoting activity in chondrocytes, but their effects on IVD cells have not been investigated yet.

**Methods:**

IVD specimens from 36 patients with spondylosis, spondylolisthesis, and scoliosis were assessed immunohistochemically for the presence of galectin‐4 and ‐8. The degrees of radiological (Pfirrmann grade) and histopathological (Rutges score) degeneration of all specimens were correlated with histological galectin scores. To assess galectin functions, separate cell cultures of annulus fibrosus (AF) and nucleus pulposus (NP) (*n* = 21) were established. Cell cultures were treated with recombinant galectin‐4, ‐8 (24 μg/mL), or Interleukin‐1β (IL‐1β) (10 ng/mL) and analyzed using RT‐qPCR and In‐Cell Western (ICW). Potential binding sites for galectins including sialylated N‐glycans and LacdiNAc structures were determined in AF and NP cells using liquid chromatography‐electrospray ionization‐tandem mass spectrometry (LC‐ESI‐MS/MS).

**Results:**

The immunohistochemical presence of galectin‐4 in IVD specimens correlated with histopathological and clinical degeneration scores of patients, whereas galectin‐8 did not show significant correlations. Both galectins were detected across IVD compartments except for the endplate. In vitro, both galectins activated the nuclear factor‐kB pathway and induced functional disease markers (Interleukin‐8 (CXCL8) and matrix metalloproteinase‐3 (MMP3) mRNA). NP cells were more responsive to galectins and IL‐1β than AF cells, indicating region‐specific differences in galectin sensitivity.

**Conclusion:**

This study identifies galectin‐4 as a novel molecular player in the pathogenesis of IVD degeneration.

## Graphical abstract

Galectin‐4 correlates with intervertebral disc degeneration, independent of age, sex, or underlying pathology. Functionally, it activates NF‐κB signaling and induces pro‐inflammatory and matrix‐degrading responses, with stronger effects in nucleus pulposus cells, identifying it as a novel molecular player in disc degeneration.

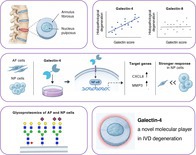

## 1 Introduction

Intervertebral disc (IVD) degeneration is the primary condition associated with low back pain, a leading cause of disability worldwide and a major contributor to the global burden of disease [1]. The IVD is a complex, avascular structure composed of three anatomically and functionally distinct regions: the cartilaginous endplate (EP), the annulus fibrosus (AF), and the nucleus pulposus (NP). Despite their close spatial proximity, these compartments are populated by distinct cell types with distinct gene expression signatures [2, 3]. For instance, Fernandes et al. demonstrated that AF cells from healthy donors predominantly express BIRC5, CCNB2, CYTL1, ESM1, and SFRP1 and show elevated expression of genes involved in the receptor signaling pathway, whereas NP cells express markers such as ANGPTL7, COL11A1, COL2A1, COL9A3, and DSC3, with upregulation of genes involved in protein biosynthesis [4]. Although various biochemical and cellular processes have been linked to the onset and progression of IVD degeneration, many aspects of its pathogenesis remain poorly understood. Moreover, effective non‐invasive treatment options are still lacking [5]. To better understand the molecular drivers of degeneration, it is essential to investigate the specific biological behavior and signaling characteristics of the different cell populations within the IVD [2, 6, 7].

One important yet underexplored feature of IVD cells is their glycophenotype—a specific set of glycan structures and glycosylation patterns on the cell surface. Glycosylation is implicated in cellular function and regulation, affecting processes such as proliferation, differentiation, apoptosis, and inflammation [8]. Galectins, a family of β‐galactoside‐binding proteins, are key modulators of glycan‐mediated signaling. They interact with cell‐surface and extracellular glycans via their carbohydrate recognition domain (CRD), enabling receptor cross‐linking and downstream signaling cascades, including transcriptional regulation [9]. Galectins are categorized into three structural types based on their CRD composition: Prototype galectins (e.g., Gal‐1, ‐2, ‐5, ‐7), which contain a single CRD and form homodimers; chimera‐type Gal‐3, which possesses a single CRD and forms multimers via an N‐terminal domain; and tandem repeat‐type galectins (e.g., Gal‐4, ‐8, ‐9), which contain two CRDs connected by a linker‐peptide, enabling multivalent interactions with glycoconjugates [8, 9]. Owing to their ability to modulate diverse cellular processes, galectins are increasingly recognized as both biomarkers and therapeutic targets in degenerative diseases [9, 10]. Dysregulation of galectins has been implicated in numerous pathological conditions, including cancer [11, 12], fibrosis [12, 13, 14], cardiovascular disorders [15, 16], and musculoskeletal degeneration. In osteoarthritis (OA), galectins—particularly Gal‐1, ‐3, ‐4, and ‐8—are overexpressed in chondrocytes and contribute to extracellular matrix breakdown and pro‐inflammatory processes [17, 18, 19, 20, 21]. Notably, these galectins appear to act cooperatively in driving OA‐associated pathomechanisms [19].

In the context of the IVD, research has primarily focused on Gal‐1 and Gal‐3 [22, 23, 24, 25]. Our previous work demonstrated that Gal‐1 is a functional driver of pro‐degenerative and pro‐inflammatory signaling in IVD cells [26], whereas Gal‐3 has been reported to decline with increasing cartilage endplate degeneration [23] and to exert protective effects against collagen damage triggered by advanced glycation endproducts [27]. Given the evidence for galectin cooperation in OA, it is plausible that other galectins—particularly those of the tandem‐repeat type—may also contribute to IVD degeneration, yet their roles remain largely unexplored. To address this knowledge gap, the present study aimed to (1) assess the presence and distribution of Gal‐4 and Gal‐8 in histological specimens of degenerated human IVDs, (2) define the glycobiological characteristics of primary AF and NP cells, and (3) examine the cellular responses of AF and NP cells to Gal‐4 and Gal‐8 stimulation. Through this approach, we sought to uncover novel insights into the glycobiological mechanisms driving IVD degeneration.

## 2 Materials and Methods

### 2.1 Clinical Specimens

Clinical specimens of the IVD were obtained from patients undergoing routine transforaminal lumbar interbody fusion. The ethics committee of the Medical University of Vienna approved the study (EK‐No.: 1720/2015, 1822/2017, 1018/2022) and all patient material was obtained with written informed consent. The medical background of the patients, including age, sex, and diagnosis, was recorded. To encompass a comprehensive range of degenerative conditions that are not solely dependent on a specific pathotype, we included patients undergoing posterior decompression and instrumented fusion for degenerative lumbar spine disease (spondylosis), isthmic spondylolisthesis with radicular symptoms (spondylolisthesis), and late correction of adolescent idiopathic scoliosis (scoliosis). Surgical indications comprised neurogenic claudication or radicular pain in spondylosis with spinal canal or foraminal stenosis, radicular symptoms, or radiographically confirmed instability in grade I–II spondylolisthesis, and curve progression or localized pain in skeletally mature patients with scoliosis.

A total of 36 histological cross sections of the IVD specimens were used for immunohistochemical staining. These included seven scoliosis patients (three females, four males) with an average age of 33.2 ± 19.7 years, eight spondylolisthesis patients (six females, two males) with an average age of 37.4 ± 9.4 years, and 21 spondylosis patients (16 females, 5 males) with an average age of 58.8 ± 11.4 years. The IVD sections were previously classified radiologically and histologically for grading of degeneration [26], confirming significantly higher mean histological degeneration scores in the spondylosis cohort compared to the spondylolisthesis and scoliosis specimens and a moderately strong correlation between the histological scores and Pfirrmann grades across all specimens. Specimens used for cell culture were separated intraoperatively into AF, NP, and EP and included a total of 21 donors (12 females, nine males; age: 21 to 85 years).

### 2.2 Galectins

Recombinant galectins, AlexaFluor‐labeled galectins, and non‐cross‐reacting antibody preparations against galectins were prepared and applied as previously described in detail [17, 19, 20, 28, 29, 30]. Galectins were fluorescently labeled by conjugating surface‐exposed lysine residues to the amino‐reactive fluorophore ALEXA488‐NHS (Invitrogen, Thermo Fisher Scientific, Dreieich, Germany) through classic N‐hydroxysuccinimidylester chemistry according to the manufacturer's instructions under activity‐preserving conditions. In line with previous studies, galectin concentrations for cell stimulation were chosen to reflect the need to overcome low intrinsic binding affinity, enable multivalent lattice formation, and account for competitive glycan interactions in vitro [19, 20, 21, 31].

### 2.3 Immunohistochemistry

Immunohistochemical staining of previously classified IVD tissue sections was performed using anti‐Gal‐4 (1:20) and anti‐Gal‐8 (1:10) as primary antibodies according to established procedures [26]. Five regions per section (EP, AF, AF/NP boundary region, NP cells and NP matrix) were graded based on the percentage of immunopositivity for Gal‐4 and Gal‐8, respectively, and staining profiles were graded from 0 to 4 according to a Labeling index = 0 (0% positivity), 1 (1%–25%), 2 (26%–50%), 3 (51%–75%), and 4 (76%–100%) [26].

### 2.4 Two‐Color Immunofluorescent Staining

Two‐color immunofluorescent staining was performed with anti‐Gal‐4 and anti‐Gal‐8 antibodies according to a previously established protocol [29]. In short, paraffin‐embedded specimens were deparaffinized and their protein‐binding sites were blocked with 10% donkey serum (Jackson Immuno Research Laboratories, West Grove, PA, USA) in 0.05 M Tris‐buffered saline (TBS). The first primary antibody (5 μg/mL anti‐Gal‐4) was applied and incubated at 4°C overnight. Subsequently, AlexaFluor488‐labeled donkey anti‐rabbit IgG (Life Technologies) was applied. Then, the sections were treated with 20% rabbit serum (Jackson Immuno Research Laboratories) in TBS and treated to prevent secondary antibody cross‐reaction with the primary rabbit antibody. Then, the second primary antibody (5 μg/mL anti‐Gal‐8S) was added and incubated overnight at 4°C before being subjected to the second secondary antibody (Alexa Fluor 555‐labeled donkey anti‐rabbit IgG; Life Technologies) and Hoechst 33258 (Sigma‐Aldrich) for nuclear staining. The colocalization was quantified with the Manders overlap coefficient using Zen software (ZEN 3.5) [32].

### 2.5 Cell Culture

AF and NP specimens were minced and transferred to cultivation flasks, facilitating outgrowth of cells. AF and NP cells were cultivated in cell culture medium (DMEM GlutaMAX (Gibco)), supplemented with 10% fetal bovine serum (Biochrom), 1% penicillin/streptomycin (Gibco), and 0.1% amphotericin B (Sigma) in a humidified hypoxic (5% CO_2_, 5% O_2_) atmosphere at 37°C. All analyses and assays were performed with cells in passage one (P1). For RT‐qPCR analysis, cells were treated with Gal‐4 or −8S (24 μg/mL) or a combination of Gal‐4 and Gal‐1 (24 + 10 μg/mL) for 24 h. In line with previous studies, galectin concentrations were selected to reflect the need to overcome low intrinsic binding affinity, enable multivalent lattice formation, and account for competitive glycan interactions in vitro [19, 20, 21, 31].

### 2.6 Glycoproteomics Sample Preparation and Glycopeptide Enrichment

Glycoproteomics samples were prepared as described previously [33]. In brief, cells were washed with phosphate‐buffered saline (PBS), pelleted by centrifugation, and lysed by adding 1 mL of 8 M urea in 100 mM Tris–HCl buffer (pH 8.0). Cysteines were reduced and alkylated by 20 mM dithioerythritol (DTT) and 40 mM iodoacetamide, respectively. Trypsin Gold (mass spectrometry grade, Promega) was added to the lysates at a protein‐to‐trypsin ratio of approximately 100:1 and digestion was carried out at 37°C for 3 h. The protein digests were purified by reversed‐phase solid‐phase extraction (C18‐SPE) cartridges (200 mg, Waters), eluted with 1.2 mL of 50% acetonitrile containing 0.05% TFA and dried using a SpeedVac concentrator. A single sample per cell line was generated by combining four biological replicates of the respective cultures. Glycopeptides were enriched using ion‐pairing hydrophilic interaction chromatography (IP‐HILIC), as described previously [34]. Dried C18‐SPE‐purified (glyco)peptides were fractionated on a TSK‐Amide 80 column (4.6 × 250 mm, 5 μm, Tosoh) developing a linear gradient from 80% acetonitrile containing 0.1% TFA to 50% acetonitrile containing 0.1% TFA over 30 min at a flow rate of 1 mL/min. Fractions (1 mL each) were collected between 25 and 39 min, resulting in 15 IP‐HILIC fractions, which were dried using a SpeedVac concentrator.

### 2.7 Glycopeptide Analysis by LC‐ESI‐MS/MS

Dried IP‐HILIC fractions were re‐suspended in 20 μL of 0.1% formic acid and subjected to LC‐ESI‐MS/MS, using a nanoEase M/Z HSS T3 column (100 Å, 1.8, 300 × 150 mm, Waters) and an Orbitrap Exploris 480 mass spectrometer (Thermo) equipped with a standard H‐ESI source operated in positive mode. Data‐dependent MS/MS analysis was performed using MS data acquired in the mass range of 350–1500 m/z at a resolution of 60 000. MS/MS data were acquired for (glyco)peptide precursor ions with charge states of 2–6 and an intensity threshold above 800 000. Stepped higher‐energy collisional dissociation (HCD) was applied with normalized collision energies of 28, 30, and 35. The first fixed mass was set to 120 m/z, and MS/MS spectra were acquired at a resolution of 30 000 in centroid mode.

### 2.8 Glycoproteomics Data Analysis and Glycopeptide Identification

Raw MS/MS data were processed using PEAKS X Pro Studio 10.6 (build 20 201 221) to extract, refine, and convert MS/MS data into the.mgf file format. Custom Perl scripts (“SugarQBits”; available at http://homepage.boku.ac.at/jstadlmann) were used for N‐glycopeptide‐specific “open‐search” analysis as reported previously [33]. Glycopeptide sequences were identified using Comet (via SearchGUI, version 4.1.15) in conjunction with a human proteome sequence database (accession number UP000005640) concatenated with reversed decoy protein sequences. Search parameters included: semi‐tryptic digestion with up to one missed cleavage, carbamidomethylation as a fixed modification (cysteines), oxidation as a variable modification (methionines), HexNAc as a variable modification (serines, threonines, asparagines). Spectral matching was performed with a mass precision of ±10 ppm (precursor) and ±0.05 amu (MS/MS). Glycopeptide identifications reported in this study were filtered to achieve an ~1% false discovery rate (FDR) at the spectrum level using the target‐decoy approach. No site localization of glycans within the peptides was performed.

### 2.9 Immunocytochemistry

AF and NP cells were cultivated on glass coverslips in a 24‐well format and stained with anti‐galectin antibodies including anti‐Gal‐1, ‐3, ‐4, ‐7, ‐8, and ‐9, and AlexaFluor488‐conjugated anti‐rabbit IgG antibody as previously described [29]. Briefly, cells were fixed with ice‐cold methanol, then rehydrated with PBS and incubated with the respective anti‐Gal antibody (20 μg/mL in 1% bovine serum albumin (BSA)/PBS) for 2 h at 37°C. After thorough washing, cells were incubated with AlexaFluor488‐conjugated anti‐rabbit IgG antibody (50 μg/mL, Thermo Fisher) and DAPI (300 nM, Sigma‐Aldrich) for 30 min. Cover slips were mounted on glass slides and imaged using a confocal laser scanning microscope (LSM 700; Carl Zeiss, Oberkochen, Germany) and Zen software.

### 2.10 RT‐qPCR

Isolation of total RNA, complementary DNA synthesis (cDNA), and SYBR green‐based qPCR measurements were performed as described before [28, 29]. In brief, treated and untreated cells were lysed, and RNA was isolated using the innuPREP RNA Mini Kit. RNA was quantified and checked for purity using the NanoDrop 2000 system before reverse transcribing to cDNA. The RT‐qPCR experiments fulfilled the minimal guidelines for the design and documentation of RT‐qPCR experiments [35]. Messenger RNA (mRNA) levels were calculated as relative quantities compared to the untreated controls (cells cultured in the same medium without addition of stimuli) and considering primer‐specific amplification efficiencies. The values were normalized to succinate dehydrogenase complex, subunit A (SDHA), which was identified as a stable reference gene under the experimental conditions used. For the establishment of relative galectin levels, the ∆Ct method was used with normalization to SDHA levels.

### 2.11 Cell‐Surface Binding

AF and NP cells were trypsinized (Trypsin–EDTA (0.25%), Gibco), and a suspension of 500 000 cells in 50 μL PBS was prepared. Following 10 min of incubation at 4°C with 0.1 μg/μl AlexaFluor488‐labeled Gal‐4 or Gal‐8 in the presence or absence of 0.1 M lactose, cells were washed with PBS and immediately imaged using a laser scanning microscope (LSM 700; Carl Zeiss, Oberkochen, Germany; Zen software).

### 2.12 In‐Cell Western

AF and NP cells were seeded in 96‐well plates and treated with 24 μg/mL Gal‐4 or Gal‐8S for 10, 60, and 180 min. To inhibit p65 phosphorylation, IKK inhibitor VII (4 μM, Merck) was added 1 h prior to the respective treatment.

Following treatment, cells were washed with PBS and fixated with ice‐cold methanol for 10 min at RT and washed again with PBS. 50 μL blocking buffer (LI‐COR Odyssey) was applied to block sites for non‐specific protein binding. The plate was incubated for 90 min on a shaking platform at RT. Primary antibodies (NF‐κB p65/mouse, Cell Signaling #6956, 1:1000 and p‐NF‐κB p65 (Ser536)/rabbit, Cell Signaling #3033, 1:800) were diluted in blocking buffer; 50 μL per well were added, and the plate was incubated overnight at 4°C. Subsequently, cells were washed with washing solution (0.1% Tween 20 in PBS) and incubated with secondary, fluorescent‐labeled antibodies (donkey anti‐mouse IgG IRDye 680RD, 1:1000 and goat anti‐rabbit IgG IRDye 800CW, 1:1000 from LI‐COR Biosciences) diluted in blocking buffer containing Tween 20 (0.2%) for 1 h at RT on a shaker platform and under light protection. After washing the cells, the plate was dried and immediately scanned using the Odyssey CLx Infrared Imaging System (LI‐COR Biosciences). The evaluation of the signals was done with the software supplied (Image Studio Version 5.2, LI‐COR Biosciences). The phosphorylation of p65 was normalized to the total amount of p65 protein and wells without treatment. Wells without cells were used as blank to eliminate background.

### 2.13 Statistics

Statistical evaluation of the data was performed using IBM SPSS v29. qPCR and ICW data were analyzed for normal distribution using the Shapiro–Wilk test, followed by pairwise comparison using the Wilcoxon signed‐rank or the Friedman test. *p* values of < 0.05 were regarded as significant. Correlations between immunopositivity scores and degeneration scores were established via Spearman correlation analyses, with *r* values being interpreted as follows: 0–0.2: weak correlation, 0.2–0.4: mild correlation, 0.4–0.6: moderate correlation, 0.6–0.8: moderately strong correlation, 0.8–1: strong correlation.

## 3 Results

### 3.1 Localization of Gal‐4 and Gal‐8 in the IVD

**FIGURE 1:**
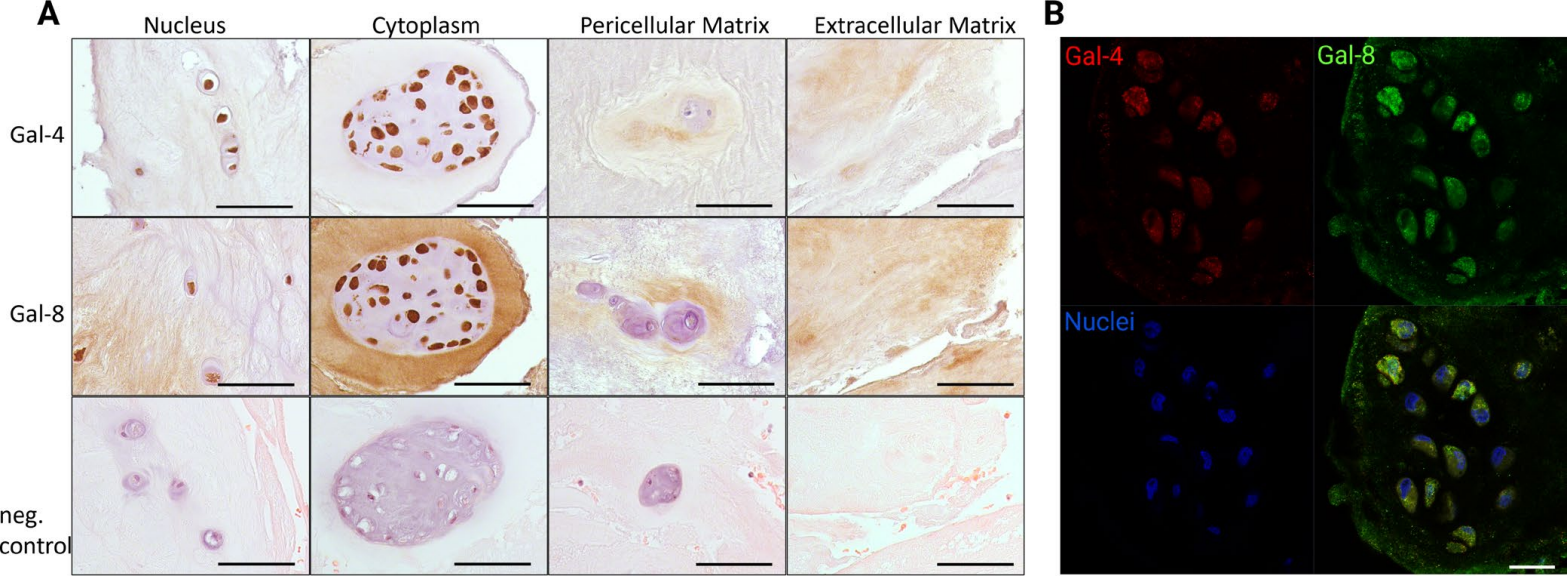
(A) Immunohistochemical localization of Gal‐4 and Gal‐8 in IVD specimens in the nucleus, cytoplasm, as well as the pericellular and extracellular matrix. Negative controls without primary antibodies are shown. Scale bars: 200 μm. (B) Immunofluorescent colocalization staining of Gal‐4 (red) and Gal‐8 (green) in IVD cell clusters. Cell nuclei are stained with DAPI and visualized in blue. Scale bars: 20 μm.

To determine whether Gal‐4 and Gal‐8 are endogenously expressed in the degenerated IVD, we performed immunohistochemical and immunofluorescent staining of histological IVD sections. First, immunohistochemistry provided evidence for the presence of Gal‐4 and Gal‐8 throughout the disc (Figure 1A). Interestingly, immunopositivity was observed in cell nuclei, cytoplasm as well as the pericellular and the extracellular matrix for both galectins to varying degrees (Figure 1A). Matrix staining was more pronounced for Gal‐8, indicating its higher abundance in extracellular compartments relative to Gal‐4. Subsequent colocalization experiments based on immunofluorescence staining of IVD sections confirmed particularly strong cytoplasmic staining for Gal‐4 (red) and Gal‐8 (green) in cells within large cell clusters (i.e., clonally expanded cells) (Figure 1B). Colocalization analysis within these clusters demonstrated a strong correlation between Gal‐4 and Gal‐8, with an overlap coefficient of 0.72, indicating that 72% of their signals coincide.

### 3.2 Immunopositivity for Gal‐4 Increases with IVD Degeneration

To investigate the clinical relevance of Gal‐4 and Gal‐8 in IVD degeneration, we assessed their immunopositivity in clinical specimens across different donor pathologies, including spondylosis, spondylolisthesis, and scoliosis. The results for Gal‐4 are presented in Table 1. The EP was negative for Gal‐4 across all cohorts. In the least degenerated IVD cohort (scoliosis), Gal‐4 staining was observed in all other anatomical regions, but not to a significantly higher extent than in the EP. In the spondylolisthesis cohort, the AF region was significantly higher immunopositive than the EP, and in the severely degenerated spondylosis cohort, immunopositivity for Gal‐4 was significantly higher in all regions than in the EP. Additionally, NP cells showed more prominent staining for Gal‐4 than AF, AF/NP, and NP matrix regions.

**TABLE 1:** Immunohistochemical scores of Gal‐4 positivity.

	Spondylosis median ± IQR	Spondylolisthesis median ± IQR	Scoliosis median ± IQR	Kruskal–Wallis test	Overall median ± IQR
EP	0 ± 0	0 ± 0	0 ± 0	*p* = 0.70^a^	0 ± 0
				*p* = 0.85^b^	
				*p* = 0.64^c^	
AF	1 ± 1	1 ± 0	1 ± 1	*p* = 0.43^a^	1 ± 1
				*p* = 0.20^b^	
				*p* = 0.45^c^	
AF/NP	2 ± 1	1 ± 0	1 ± 0	*p* = 0.11^a^	1 ± 1
				*p* = 0.08^b^	
				*p* = 0.56^c^	
NP cells	2 ± 2	1 ± 1	2 ± 2	*p* = 0.14^a^	2 ± 2
				*p* = 0.83^b^	
				*p* = 0.25^c^	
NP matrix	1 ± 2	0.5 ± 1	0 ± 1	*p* = 0.25^a^	1 ± 1
				*p* = 0.11^b^	
				*p* = 0.69^c^	
Total Score	7 ± 4.5	4.5 ± 2	5 ± 5	*p* = 0.10^a^	5 ± 4
				*p* = 0.28^b^	
				*p* = 0.73^c^	
Friedman test					
NP cells vs. EP	*p* < 0.01				*p* < 0.01
NP cells vs. AF	*p* < 0.01				*p* < 0.01
NP cells vs. AF/NP					*p* < 0.01
NP cells vs. matrix	*p* < 0.01				*p* < 0.01
NP matrix vs. EP	*p* < 0.01				*p* < 0.01
AF vs. EP	*p* < 0.01	*p* < 0.01			*p* < 0.01
AF/NP vs. EP	*p* < 0.01				*p* < 0.01

*Note:* The median values (± Interquartile range IQR) of Gal‐4 positivity scores for EP, AF, AF/NP boundaries, NP cells, NP matrix, and Total score for all three cohorts and overall median values (± IQR) are shown. In addition, the *p* values of the Kruskal–Wallis test between the three cohorts and the Friedman test (with pairwise comparison and Bonferroni correction) within a cohort for the anatomical regions of the IVD are presented.

^a^ Spondylosis versus Spondylolisthesis.

^b^ Spondylosis versus Scoliosis.

^c^ Spondylolisthesis versus Scoliosis.

**FIGURE 2:**
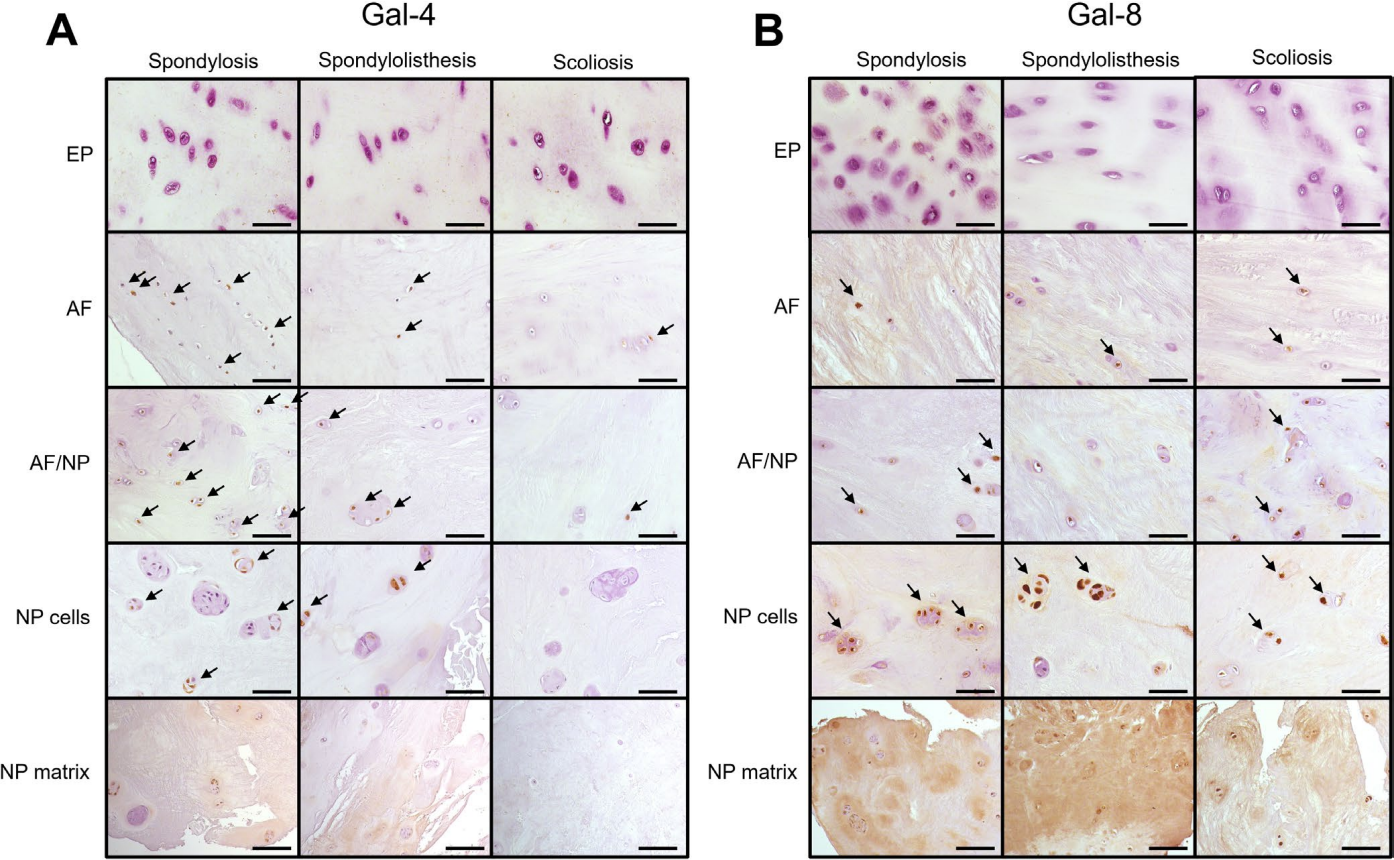
Immunohistochemical staining profiles of (A) Gal‐4 positivity and (B) Gal‐8 positivity across all regions (EP, AF, AF/NP, NP cells and NP matrix) of the three cohorts (Spondylosis, Spondylolisthesis and Scoliosis). Arrows indicate galectin‐positive cells. Scale bars EP, AF, AF/NP, NP cells = 50 μm, NP matrix = 200 μm.

It is noteworthy that no significant differences were observed between the cohorts, suggesting that the underlying pathology itself was not a distinguishing feature. Nevertheless, it became obvious that, across all specimens, Gal‐4 immunopositivity was predominant in strongly degenerated samples of all cohorts, particularly in NP cells. This observation gave rise to the hypothesis that the degree of degeneration in the IVD specimens was a contributing factor for Gal‐4 immunopositivity (Figure 2A).

**FIGURE 3:**
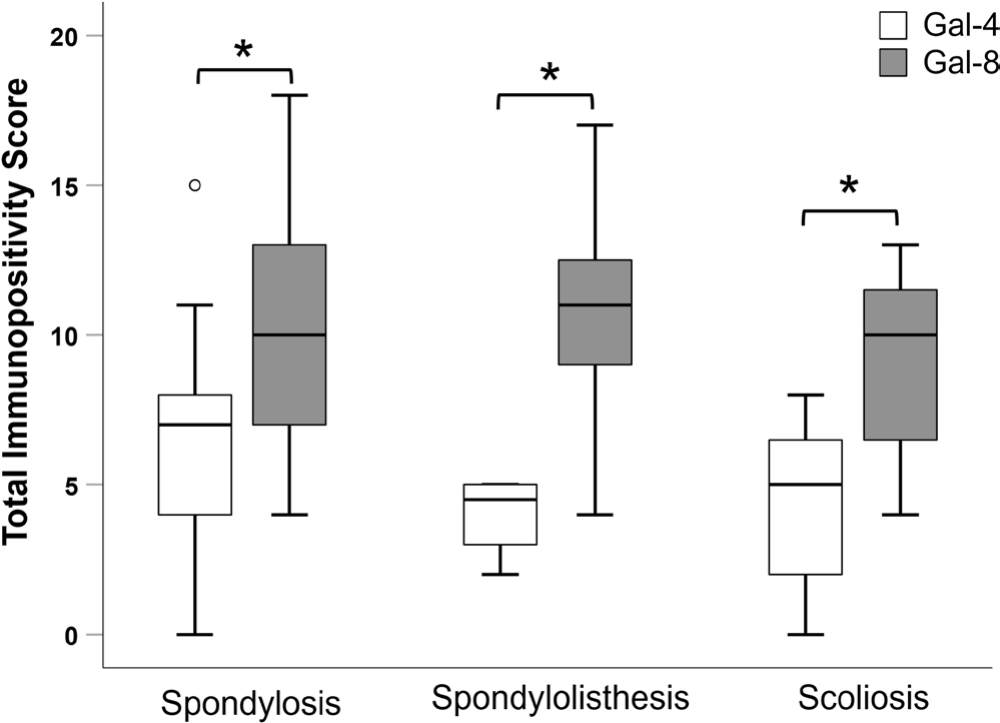
Total scores of Gal‐4 and Gal‐8 per cohort are shown as box plots (median, interquartile range, and whiskers indicating minimum and maximum values) (Spondylosis *p* < 0.001; Spondylolisthesis *p* = 0.012; Scoliosis *p* = 0.018). Significance evaluated by Wilcoxon signed rank test.

The immunohistochemical scores of Gal‐8 positivity in the different regions of the IVD specimens across all three cohorts (spondylosis, spondylolisthesis, and scoliosis) are presented in Table 2. As for Gal‐4, the total scores of Gal‐8 immunopositivity showed no difference between the cohorts. Interestingly, the total scores of Gal‐8 were significantly higher across all cohorts than the Gal‐4 immunopositivity (Figure 3). This was also confirmed for all separate regions (data not shown). In the spondylosis cohort and overall, Gal‐8 immunopositivity was higher in all regions than in the EP, but in contrast to Gal‐4, there was no significant difference between NP cells and AF, AF/NP border region, or NP matrix. The strongest staining intensity was found in the NP matrix (Figure 2B).

**TABLE 2:** Immunohistochemical scores of Gal‐8 positivity.

	Spondylosis median ± IQR	Spondylolisthesis median ± IQR	Scoliosis median ± IQR	Kruskal–Wallis test	Overall median ± IQR
EP	0 ± 1	1 ± 1	1 ± 1	*p* = 0.63^a^	1 ± 1
				*p* = 0.29^b^	
				*p* = 0.56^c^	
AF	2 ± 2	3 ± 1	2 ± 2	*p* = 0.32^a^	2 ± 2
				*p* = 0.65^b^	
				*p* = 0.25^c^	
AF/NP	2 ± 2	3 ± 2	2 ± 3	*p* = 0.75^a^	2 ± 2
				*p* = 0.96^b^	
				*p* = 0.86^c^	
NP cells	3 ± 2	2 ± 2.5	3 ± 2	*p* = 0.53^a^	3 ± 2
				*p* = 0.46^b^	
				*p* = 0.95^c^	
NP matrix	3 ± 2.5	3.5 ± 2	1 ± 2	*p* = 0.42^a^	3 ± 3
				*p* = 0.16^b^	
				*p* = 0.09^c^	
Total Score	10 ± 7	11 ± 3.5	10 ± 8	*p* = 0.63^a^	10 ± 5.5
				*p* = 0.63^b^	
				*p* = 0.49^c^	
Friedman test					
EP vs. AF	*p* < 0.01	*p* < 0.01			*p* < 0.01
EP vs. AF/NP	*p* < 0.01				*p* < 0.01
EP vs. NP cells	*p* < 0.01				*p* < 0.01
EP vs. NP matrix	*p* < 0.01	*p* < 0.01			*p* < 0.01

*Note:* The median values (± IQR) of Gal‐8 positivity scores for EP, AF, AF/NP boundaries, NP cells, NP matrix, and Total score for all three cohorts and overall median values (± IQR) are shown. In addition, the *p* values of the Kruskal–Wallis test between the three cohorts and the Friedman test (with pairwise comparison and Bonferroni correction) within a cohort for the anatomical regions of the IVD are presented.

^a^ Spondylosis versus Spondylolisthesis.

^b^ Spondylosis versus Scoliosis.

^c^ Spondylolisthesis versus Scoliosis.

**FIGURE 4:**
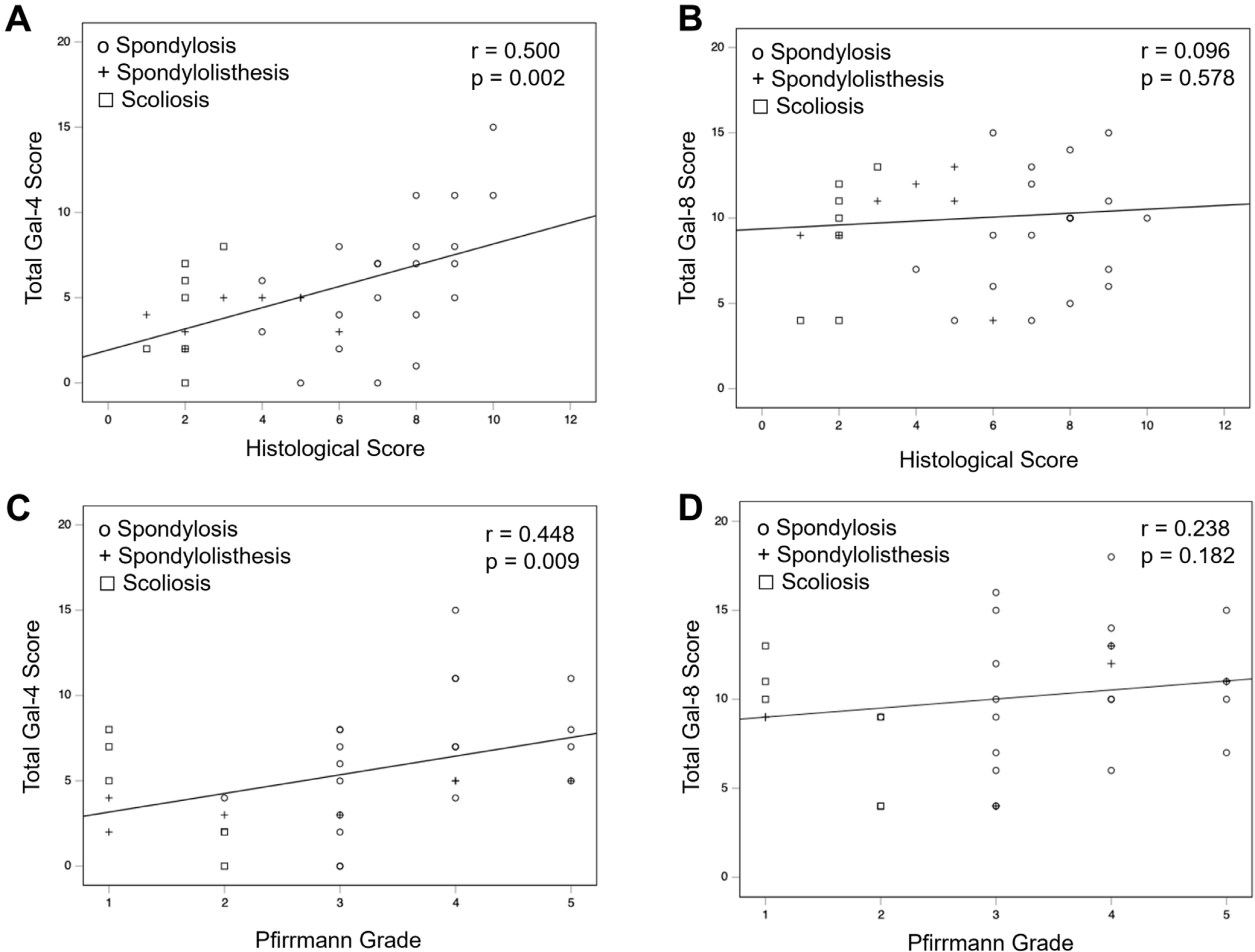
(A) Scatterplot of correlation of total Gal‐4 score versus histological degeneration (Rutges scale). (B) Scatterplot of correlation of total Gal‐8 versus histological degeneration (Rutges scale). (C) Scatterplot of correlation of total Gal‐4 score versus radiologic degeneration (Pfirrmann grade). (D) Scatterplot of correlation of total Gal‐8 versus radiologic degeneration (Pfirrmann grade). Regression line, Spearman correlation coefficient *r*, and *p* value (bivariate correlation test) are given.

To further explore the hypothesized relationship between galectins and IVD degeneration, we correlated total Gal‐4 and Gal‐8 scores with the histopathological degeneration obtained using the Rutges scale. The analysis revealed a moderate positive correlation between total Gal‐4 scores and the Rutges scale (*r* = 0.5, *p* < 0.01) (Figure 4A), whereas no significant correlation was found for total Gal‐8 scores (*r* = 0.096, *p* = 0.578) (Figure 4B). Expanding this analysis to the clinical context, we examined correlations of total Gal‐4 or Gal‐8 scores with the radiological degeneration of the IVDs as indicated by the Pfirrmann grade. Similarly, Gal‐4 showed a moderate positive correlation with the Pfirrmann grade (*r* = 0.448, *p* < 0.01) (Figure 4C), while Gal‐8 levels remained unchanged (*r* = 0.238, *p* = 0.182) (Figure 4D). These findings reinforce the previous observation that Gal‐4 immunopositivity was associated with histopathological degeneration, whereas Gal‐8 remained unaffected. In our previous study on Gal‐1 and Gal‐3 performed on the same specimen set using identical methodology [25], we had identified a positive correlation between Gal‐1 and both histopathological and radiological degeneration scores (figure 1 in [25]). To investigate a potential interaction between Gal‐1 and Gal‐4, we performed a correlation analysis, which revealed a moderate positive correlation (*r* = 0.551, *p* < 0.001), suggesting a cooperative role of these galectins in IVD degeneration. Next, we examined the relationship between galectin immunopositivity and patient‐related factors. Given the broad donor age range in our IVD sample set and previous reports linking certain galectins to aging [33, 34], we first analyzed correlations between total Gal‐4 and Gal‐8 levels and donor age. However, in our sample set, no significant correlations were found for Gal‐4 (*r* = 0.087, *p* = 0.614) or Gal‐8 (*r* = −0.076, *p* = 0.66). Likewise, no association with sex was observed for total Gal‐4 (*r* = −0.2, *p* = 0.25) and Gal‐8 (*r* = −0.118, *p* = 0.50) levels.

### 3.3 Characterization of AF and NP Cell Populations

Next, we investigated the molecular mechanisms induced by Gal‐4 and Gal‐8 in the context of IVD degeneration. To elucidate the function of galectins in different IVD compartments, we intraoperatively separated human IVDs into AF and NP regions and established primary cultures of AF and NP cells. Successful separation of AF and NP cells was confirmed by measuring the mRNA expression levels of three established NP marker genes (COL2A1, COMP, ACAN [4]) using RT‐qPCR in samples from 15 donors. Comparative analysis revealed significantly higher COL2A1 expression in NP cells (mean = 0.039 ± 0.088) compared to AF cells (mean = 0.0074 ± 0.015; *p* = 0.002). Similarly, COMP expression was significantly higher in NP cells (mean = 5.27 ± 4.93) than in AF cells (mean = 2.40 ± 3.23; *p* = 0.004), while ACAN expression showed a trend toward elevated levels in NP cells (*p* = 0.053).

To further characterize these cell populations, particularly regarding their glycophenotype, we quantified the mRNA levels of various glycosyltransferases using RT‐qPCR (Table S1). The mRNA profile indicates the presence of an enzymatic machinery that is responsible for the synthesis of complex N‐glycans (MAN2, MGAT2) with fucose core substitutions (FUT8) and branching (MGAT4B, MGAT5). Expected are also O‐glycans as GALNT initiates O‐linked glycosylation. B3GNT2 and GCNT suggest the production of core 2 glycans with LacNAc repeats. Sialylation is also likely in both glycan types. Overall, these data suggest that all essential requirements for glycan biosynthesis are present in AF and NP cells. To further elucidate the protein glycosylation landscape of AF and NP cells, we analyzed their glycoproteomes by LC‐ESI‐MS/MS.

### 3.4 Glycoproteomics Analysis

Comparative analysis of AF and NP cells revealed highly similar glycoproteomes of the two cell populations (Data S1). Both datasets are vastly dominated by a small number of highly abundant, mostly extracellular glycoproteins, including FN1, COL6A, COL1A, and THBS1, as well as cell‐surface‐associated glycoproteins CD63, CD59, LAMP1, LAMP2, ANPEP, and PSAP, which all exhibited vastly overlapping glycosylation‐site‐specific glycosylation profiles in AF and NP cells.

**FIGURE 5:**
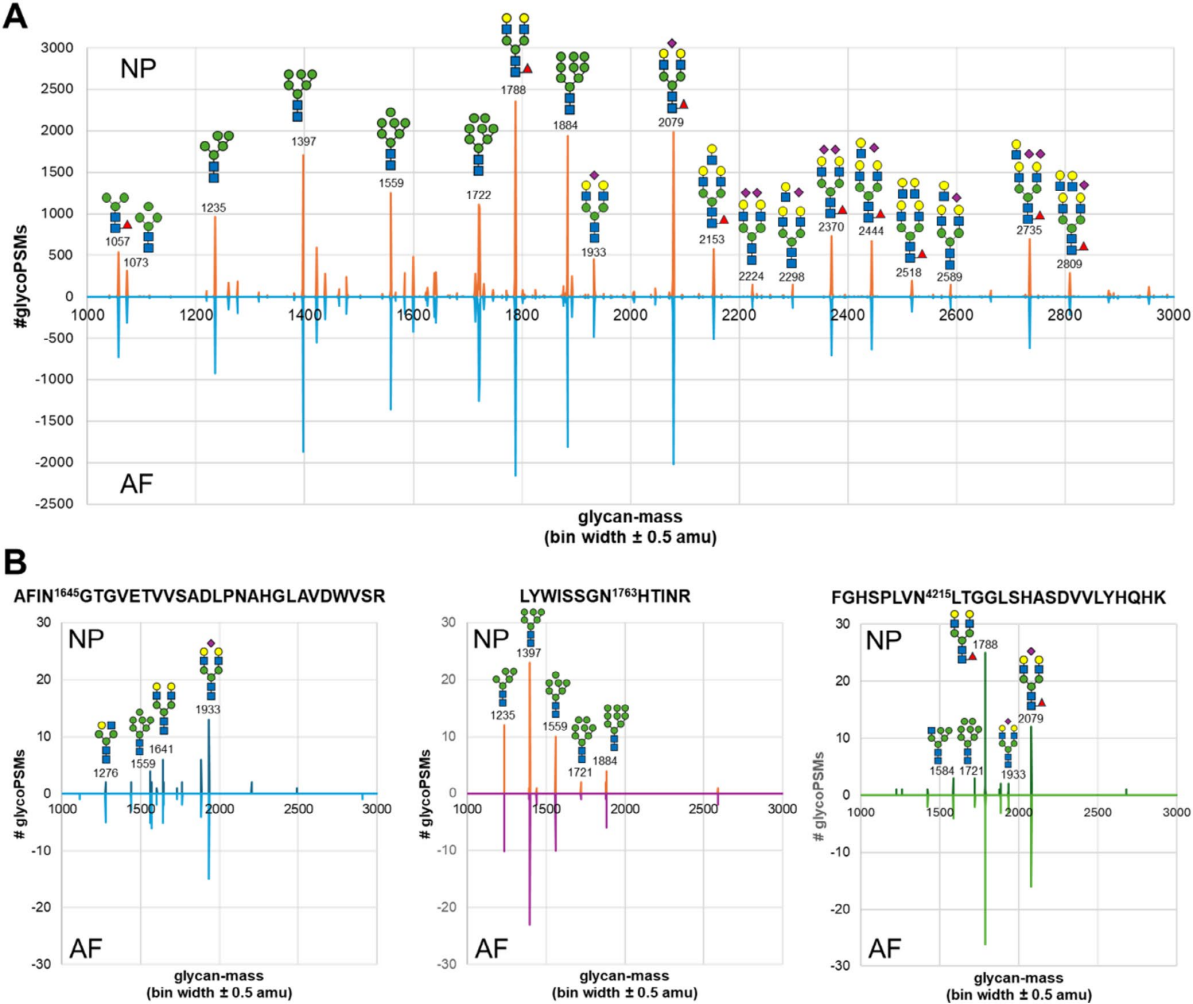
Glycoproteomic profiling of annulus fibrosus (AF) and nucleus pulposus (NP) cells. (A) Whole‐cell lysates were analyzed by LC–ESI–MS/MS using data‐dependent acquisition, and glycopeptides were identified with a glyco‐focused open‐search workflow (SugarQbits [33]). Semi‐quantitative glycan mass distributions were reconstructed from spectral counts of the mass difference between each precursor and its Y1 ion (peptide + HexNAc) and plotted as histograms (bin width: 1 Da). (B) Site‐specific comparisons within low‐density lipoprotein receptor‐related protein 1 (LRP1; UniProt Q07954) showed that Asn1645 predominantly carried non‐fucosylated complex‐type N‐glycans, Asn1763 was exclusively modified with oligomannose‐type N‐glycans, and Asn4215 predominantly carried fucosylated N‐glycans. Cartoon annotations indicate tentative glycan structures inferred from mass‐based compositions and are rendered using the Symbol Nomenclature for Glycans (SNFG) [36].

Similarly, and in line with the mRNA levels of glycosyltransferases determined by RT‐qPCR, a semiquantitative comparison of all N‐glycan masses detected in NP and AF cells (Figure 5A) showed almost identical glycome structures, comprising oligomannose‐type (i.e., Man3‐Man9), hybrid‐type and core‐fucosylated, sialylated complex‐type N‐glycans, with multiple antennae and/or terminal LacNAc (i.e., GlcNAc‐beta1,4‐Gal) repeating units. This finding was corroborated by site‐specific glycosylation analysis using low‐density lipoprotein receptor‐related protein 1 (LRP1) as a representative example, which showed comparable site occupancy and glycan composition in AF and NP cells (Figure 5B), with LRP1 having recently been implicated in intervertebral disc degeneration [37]. Of note, potential differences in the linkage‐type of sialic acid residues (i.e., alpha‐2,6 or alpha‐2,3) between AF and NP cells (as would, e.g., be suggested from different mRNA levels of ST3Gal4 in the two cell populations) could not be resolved by this analytical approach.

### 3.5 Galectin Profile

To examine native galectin expression in isolated and cultured IVD cell subpopulations, we performed immunocytochemical staining of AF and NP cells using various anti‐galectin antibodies, confirming galectin presence (Figure S1). To provide a quantitative assessment, we measured mRNA expression levels of LGALS1, 2, 3, 4, 7, 8, and 9 in AF and NP cells (Table S2). Among all galectins, LGALS1 exhibited the highest mRNA expression in both cell populations (~55 000‐fold relative to SDHA), followed by LGALS3 (~4500‐fold) and LGALS8 (~300‐fold). Consistent with our histological findings (Figure 2), LGALS4 expression (~3‐fold) was significantly lower than that of LGALS8 (*p* = 0.023). Notably, LGALS2 and LGALS3 expression differed significantly between NP and AF cells: LGALS2 was more highly expressed in NP cells (*p* = 0.046), whereas LGALS3 was more prominently expressed in AF cells (*p* = 0.028).

### 3.6 Gal‐4 and Gal‐8 Bind to AF and NP Cell Surfaces

**FIGURE 6:**
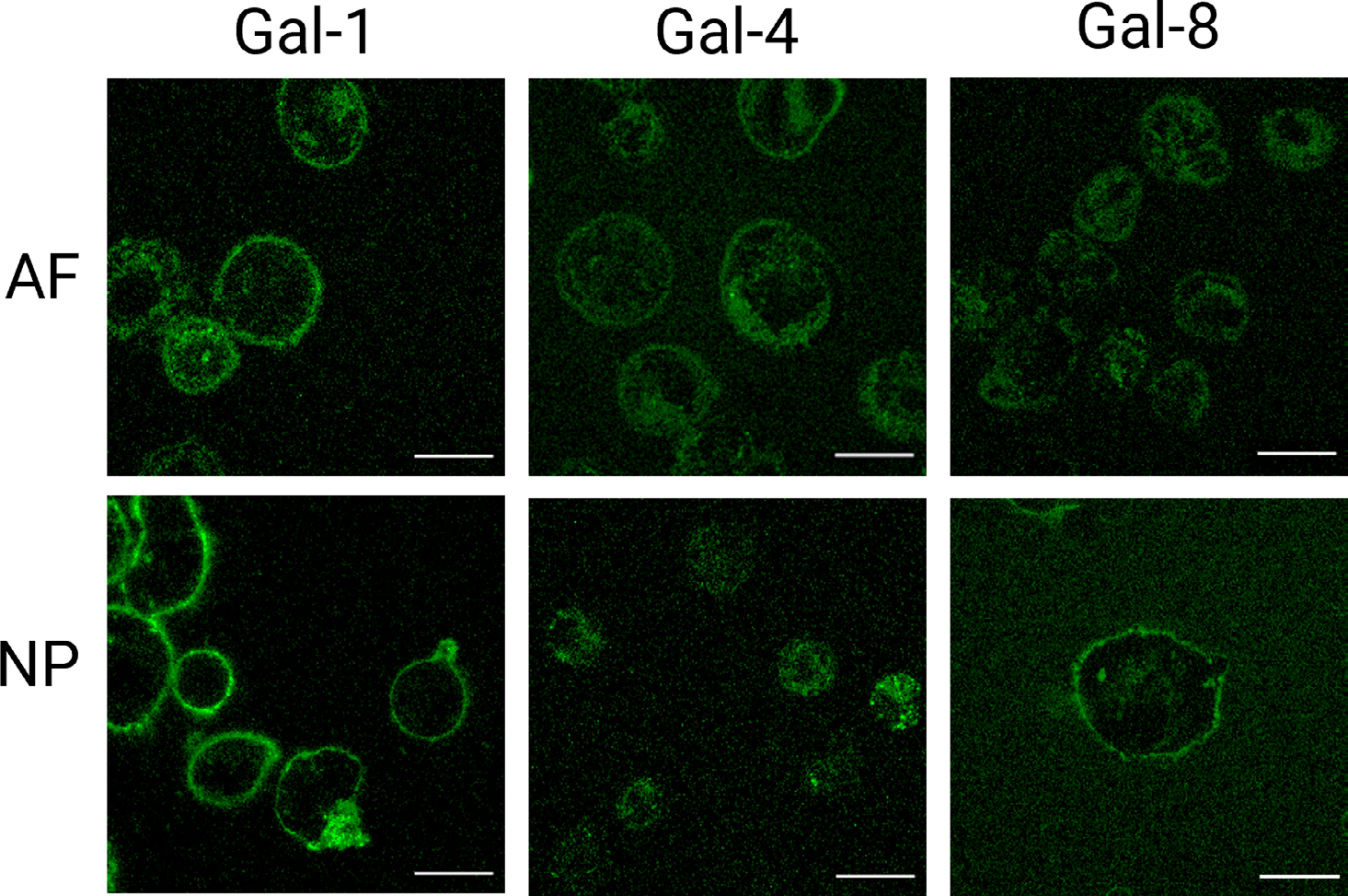
Localization of AlexaFluor488‐marked Gal‐1, Gal‐4, and Gal‐8 upon addition at 4°C to viable single cells of the AF and NP. Cells were trypsinized and subjected to the fluorescent‐labeled galectins for 10 min. Images captured and analyzed using a Carl Zeiss LSM700 at 630 × magnification. Scale bars: 20 μm.

After characterizing NP and AF cells, we next investigated the potential of extracellular galectin to bind their cell surfaces. Thus, we incubated fluorescence‐labeled Gal‐4 and Gal‐8 with NP and AF cells at 4°C to inhibit cellular uptake (Figure 6). Gal‐1 served as a positive control, showing the expected strong binding to cell surfaces. Compared to Gal‐1, both Gal‐4 and Gal‐8 showed reduced—but still evident—binding to AF and NP cell surfaces at 4°C.

### 3.7 Gal‐4 and Gal‐8 Trigger the NF‐ҡB Signaling Pathway in a Time‐Dependent Manner

**FIGURE 7:**
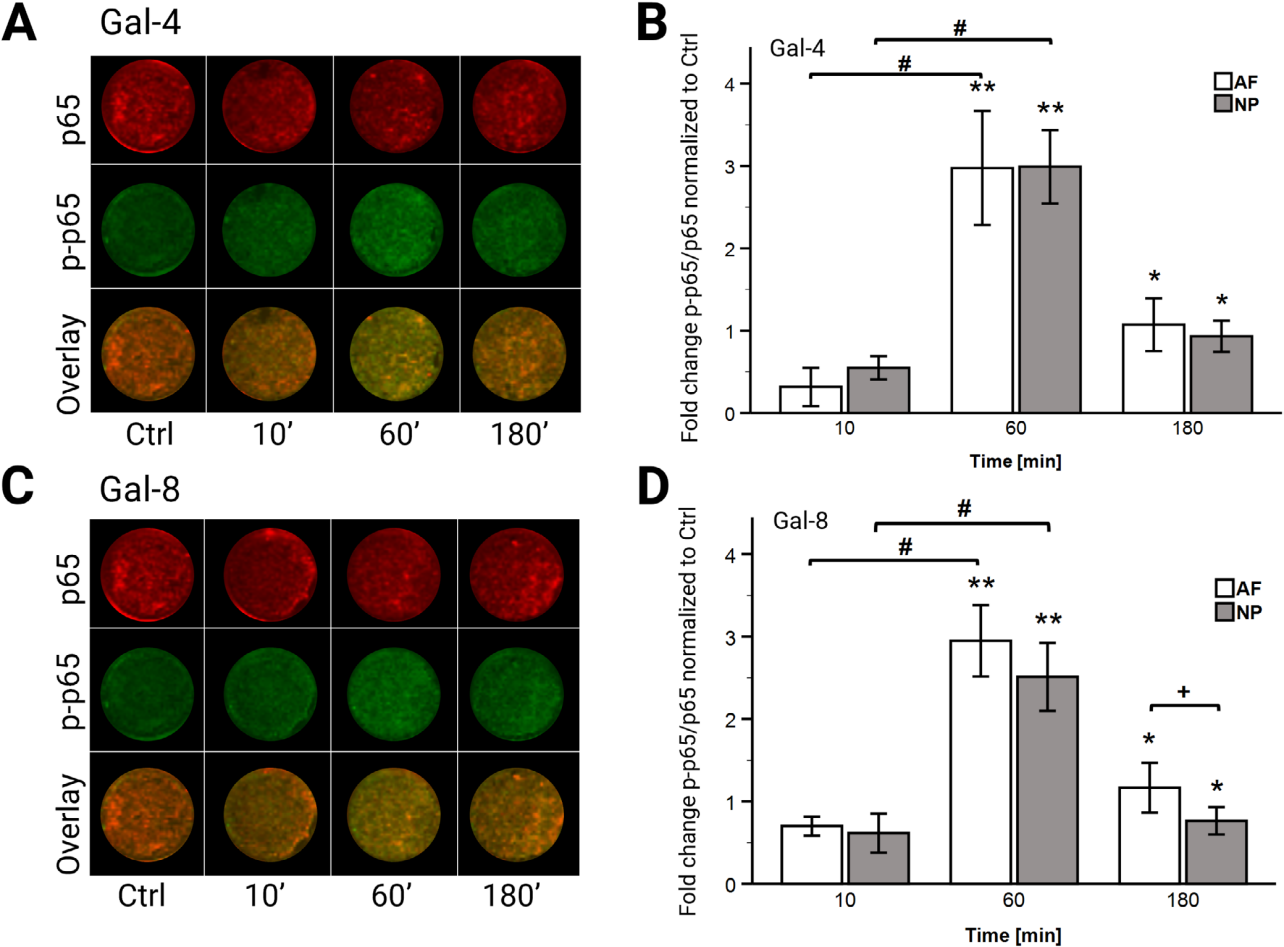
AF and NP cells from four donors (*n* = 4) were treated with 24 μg/mL Gal‐4 (A–B) or 24 μg/mL Gal‐8 (C–D) over time, and NF‐κB activation was measured by In‐Cell Western (2 technical replicates). (A) A representative scan of NP cells from one donor treated with Gal‐4 is shown. Control cells represent cells cultured in medium without addition of the respective stimuli. (B) Signal intensities of phosphorylated p65 were normalized to total p65 and shown as mean values and standard deviations (*n* = 4). Significant differences to untreated control cells (cultured in medium without addition of the respective stimuli; set to 1) are indicated with asterisks (*p < 0.05, **p < 0.001, Friedman pairwise comparison); significant differences between timepoints are indicated with hash (#*p* < 0.05, Friedman pairwise comparison). (C) One representative scan of NP cells treated with Gal‐8 is shown. Control cells represent cells cultured in medium without addition of the respective stimuli. (D) Signal intensities of phosphorylated p65 were normalized to total p65 and shown as mean values and standard deviations (*n* = 4). Significant differences to untreated control cells cultured in medium without addition of the respective stimuli; set to (1) are indicated with asterisks (*p < 0.05, **p < 0.001, Friedman pairwise comparison); significant differences between timepoints are indicated with hash (#*p* < 0.05, Friedman pairwise comparison); and significant differences between cell populations are indicated by a plus (+ *p* < 0.05, Wilcoxon).

In‐cell Western analyses were used to investigate galectin‐induced signaling mechanisms. Given the role of NF‐ҡB signaling in galectin‐mediated cartilage degeneration [17], we compared p65‐phosphorylation in AF and NP cells at various time points after treatment with Gal‐4 or Gal‐8, using untreated cells as control. As shown in Figure 7, both galectins induced a time‐dependent phosphorylation of p65 in AF and NP cells, peaking at 60 min (3‐fold increase) and remaining significantly elevated at 180 min. Except for Gal‐8 at 180 min, no significant differences were observed between NP and AF cells. These findings suggest that Gal‐4 and Gal‐8 activate the NF‐ҡB pathway in vitro, potentially leading to upregulation of degenerative and inflammatory marker genes.

### 3.8 Galectin Sensitivity Varies Between AF and NP Cells

**FIGURE 8:**
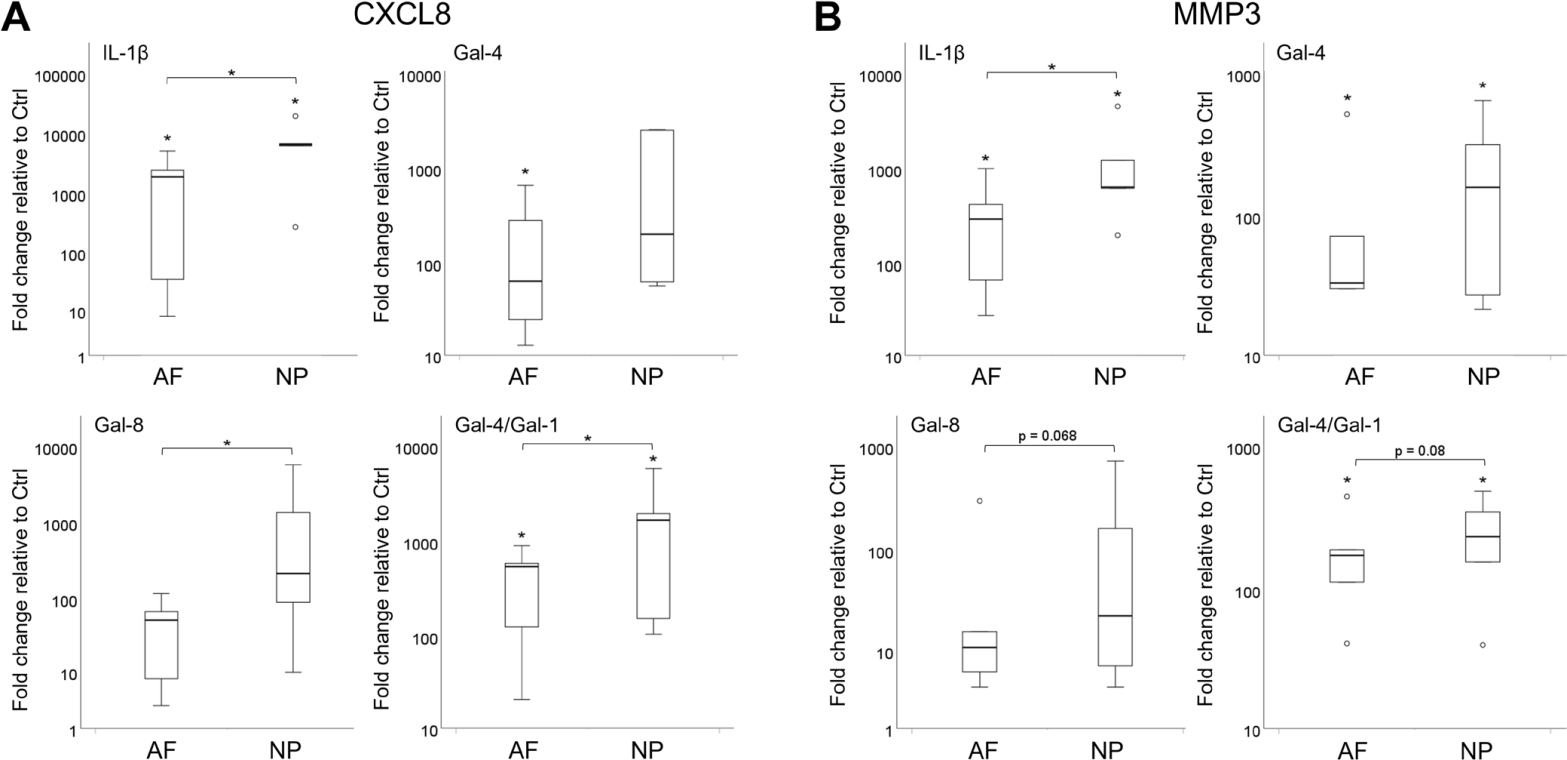
(A) Relative mRNA expression of CXCL8 of AF and NP cells (*n* = 5) after treatment with IL‐1β (1 ng/mL), Gal‐4, Gal‐8 (24 μg/mL), and Gal‐4/Gal‐1 (24/10 μg/mL) combination. (B) Relative mRNA expression of MMP3 of AF and NP cells (*n* = 5) after treatment with IL‐1β, Gal‐4, Gal‐8, and Gal‐4/Gal‐1 combination. Fold changes are shown relative to untreated controls (cells cultured in medium without addition of the respective stimuli). The *p* values of individual cell types were calculated with Friedman's pairwise comparison, and the comparison of cell types was calculated using the Wilcoxon signed‐rank test.

In a next step, we investigated the reactivity of the two IVD cell populations (AF and NP cells) to Gal‐4, Gal‐8, IL‐1β, and a mixture of Gal‐4 and Gal‐1. We measured the relative mRNA expression of pro‐inflammatory (CXCL8) and degenerative (MMP3) marker genes in both cell populations (*n* = 5 patients). Evaluation of the inflammatory response using CXCL8 revealed that both cell populations were highly susceptible to all stimuli (Figure 8A). IL‐1β treatment significantly increased CXCL8 expression in both cell types, with NP cells showing a 3.5‐fold higher upregulation of CXCL8 compared to AF cells.

After treatment with Gal‐4, the AF population exhibited a significant 65‐fold upregulation of CXCL8 (*p* = 0.046), while NP cells showed a 205‐fold increase compared to the respective untreated cells. Although this was not statistically significant, the data suggest a trend toward higher reactivity in NP cells. Given the results of our previous study [26], which showed a positive correlation between Gal‐1 levels and IVD degeneration, as well as the enhancing effects of Gal‐1 on inflammatory and degenerative marker genes, we combined Gal‐1 and Gal‐4 to assess their cooperative effects. The combination of Gal‐1 and Gal‐4 produced a significant 553‐fold increase in CXCL8 expression in AF cells (*p* = 0.001) and an even higher 1700‐fold upregulation in NP cells (*p* = 0.043). In contrast, treatment with Gal‐8 did not significantly increase CXCL8 expression in either AF or NP cells. However, NP cells exhibited a significantly higher susceptibility (216‐fold increase) compared to AF cells (52‐fold increase).

Similarly, the mRNA expression of MMP3 as a degenerative marker (Figure 8B) showed a trend consistent with the pro‐inflammatory response. IL‐1β treatment significantly upregulated MMP3 in both cell types, with NP cells being more reactive than AF cells. Gal‐4 treatment induced a significant 162‐fold upregulation of MMP3 in NP cells, whereas the increase in AF cells was markedly lower (34‐fold). The combination of Gal‐4 and Gal‐1 led to a significant increase in MMP3 expression, with a 175‐fold increase in AF cells and a 237‐fold increase in NP cells. Again, NP cells showed a pronounced trend toward higher reactivity (*p* = 0.08). Treatment with Gal‐8 also revealed a trend toward higher reactivity of NP cells (23‐fold, *p* = 0.068), but there was no significant upregulation of MMP3 expression in either cell population.

In summary, both AF and NP cells are susceptible to treatments with galectins and IL‐1β, with NP cells demonstrating greater reactivity. Gal‐8 appears to elicit less reactivity than Gal‐4, and the combination of Gal‐4 and Gal‐1 produces additive effects.

## 4 Discussion

This study provides new mechanistic and translational insights into the function of Gal‐4 in IVD degeneration. While Gal‐8 has been implicated more prominently in degenerative processes in other tissues such as articular cartilage [19], our data identify Gal‐4 as a galectin strongly associated with IVD degeneration, particularly within the NP cellular compartment.

Immunohistochemical analysis revealed that Gal‐4 expression positively correlated with both histopathological and radiological degeneration grading, whereas Gal‐8 was present across all regions and degeneration grades without a clear association. Neither age nor sex correlated with Gal‐4 or Gal‐8 positivity in this study, suggesting that Gal‐4 may serve as a tissue‐intrinsic and degeneration‐specific biomarker of the IVD, independent of demographic variables. This observation parallels our previous findings on Gal‐1 in human IVD tissue [22, 26]. Interestingly, both Gal‐4 and Gal‐8 were scarcely detectable in the cartilaginous EP, consistent with earlier observations for Gal‐1 and Gal‐3 [26]. This pattern may reflect the relative structural stability and delayed degeneration of the EP, or alternatively a reduced involvement of galectin‐mediated signaling pathways in this region compared to the AF and NP, which are more vulnerable to structural and biochemical deterioration [26]. When integrating the present findings with our previous study on Gal‐1 and ‐3 in the same specimen set, a more differentiated picture of galectin involvement in IVD degeneration emerges. While Gal‐1 and Gal‐4 both show robust correlations with degeneration independent of age, Gal‐3 appears to be additionally influenced by demographic factors, and galectin‐8 does not exhibit a clear association with degeneration severity.

A major strength of this study is the use of well‐characterized primary human AF and NP cells from surgical specimens, enabling the investigation of cell type‐specific galectin interactions and downstream signaling responses. While earlier studies have begun to distinguish AF and NP cell populations [4, 6, 38], few have combined functional assays with surface‐focused glycophenotyping approaches [39]. Here, we showed that both Gal‐4 and Gal‐8 bind to the surface of AF and NP cells. Subsequent glycoproteomic analysis identified several putative galectin ligands previously linked to IVD degeneration and galectin biology. For example, CD63, a tetraspanin, has been proposed as a potential therapeutic target in IVD degeneration [40]. Given its glycosylation and localization within tetraspanin‐enriched microdomains, CD63 may function as part of galectin–glycan interaction networks that regulate receptor clustering, membrane organization, and cellular signaling [41]. LAMP1 and LAMP2, which are involved in lipophagy and autophagy, show altered expression in degenerated IVD tissues [42, 43], and have been reported to interact with galectins, particularly Gal‐3 and Gal‐8 [44, 45, 46, 47]. CD59, an inhibitor of the terminal complement complex—an inflammatory trigger and cause of cell lysis—has been shown to correlate positively with the severity of IVD degeneration [48]. Moreover, CD59 is linked to clathrin‐independent endocytosis, and its uptake is stimulated by Gal‐3 interactions [49]. In addition, ANPEP emerged as one of the top 20 upregulated proteins in extracellular vesicles derived from degenerated IVDs [50] and has previously been associated with Gal‐3 [51]. PSAP has also been suggested to promote the exosomal secretion of Gal‐3 [11]. Together, these findings support the concept that galectins engage a conserved but disease‐modulated surface interactome in human IVD cells.

Functionally, both Gal‐4 and Gal‐8 induced p65 phosphorylation, implicating NF‐κB signaling as a shared downstream pathway. However, Gal‐4 elicited a more robust pro‐degenerative and pro‐inflammatory transcriptional response, significantly increasing the expression of MMP3 and CXCL8, particularly in NP cells. Extending this comparison to our previous work on Gal‐1 in the same specimen set, a consistent pattern emerges: all galectins studied so far engage NF‐κB signaling, yet differ in their potency and downstream effects: Gal‐1 and Gal‐4 induce pronounced pro‐inflammatory and matrix‐remodeling responses, whereas Gal‐8 shows more moderate effects despite similar pathway activation. The enhanced NP cell reactivity may be attributable to their notochordal origin [2, 52], which may confer an increased sensitivity to inflammatory or stress‐related cues. Given the comparable glycoprotein profiles between AF and NP cells—a finding that is in line with a previous report on the glycan level [39]—these differential responses point to additional regulatory layers downstream of ligand binding, potentially involving cell type‐specific signal transduction mechanisms or differences in glycan fine structure, such as sialic acid linkage patterns, which were not assessed in the present study. Future linkage‐specific glycomic analyses will be essential to resolve these mechanisms.

Based on previous reports of cooperative galectin activity in cartilage degeneration [19], we also explored potential synergistic effects between Gal‐4 and Gal‐1. Combined stimulation of AF and NP cells with both galectins induced significantly stronger degenerative and pro‐inflammatory responses than Gal‐4 alone, suggesting functional cooperation within a galectin network. These findings are particularly relevant in light of the concurrent upregulation of multiple galectins in degenerative tissues and may have implications for therapeutic strategies aiming to modulate galectin signaling rather than targeting individual family members in isolation. Together, these results support a model of coordinated, partially overlapping galectin activities rather than functional redundancy within the degenerating IVD.

As with any in vitro study, several limitations must be acknowledged. Monoculture systems lack the cellular, structural, and biomechanical complexity of native IVD tissue, and cell expansion can induce phenotypic drift. We minimized these effects by using passage 1 cells and avoiding enzymatic tissue digestion during cell isolation. However, tissue outgrowth may preferentially select for more migratory, fibroblast‐like cell populations, which could influence the cellular composition and thereby impact the study outcome. Moreover, the absence of cell–cell or matrix‐cell interactions in the used cell culture models limits direct translation. Further studies employing 3D co‐culture, biomimetic scaffolds, or organotypic IVD models will be essential to validate these findings in a more physiologically relevant context [53]. In addition, the integration of glycomics with functional assays will be critical to define the specific glycan determinants that govern galectin binding and signaling in IVD cells.

## 5 Conclusion

This study identifies Gal‐4 as a previously unrecognized molecular contributor to the pathogenesis of human IVD degeneration. Gal‐4 expression strongly correlated with degeneration severity and was predominantly localized to the NP, where it induced pro‐inflammatory and matrix‐degrading responses in primary human IVD cells. Importantly, these effects were more pronounced than those triggered by Gal‐8, and NP cells exhibited heightened sensitivity to Gal‐4 stimulation. Despite largely similar glycoproteomic profiles between NP and AF cells, the observed differential cellular responses suggest cell‐type‐specific downstream signaling or glycan‐based regulatory mechanisms that warrant further investigation. Collectively, these findings have potential clinical relevance, as Gal‐4 appears to function not only as a marker of disease severity but also as an active driver of the degenerative phenotype. The observed additive effects with Gal‐1 further support the concept of cooperative galectin networks in disc pathology. Targeting Gal‐4, either alone or in combination with other galectins, may therefore represent a promising strategy to modulate inflammation and matrix degradation in IVD degeneration. Future studies using advanced 3D or organotypic models will be critical to validate these effects under more physiological conditions and to explore translational therapeutic approaches.

## Author Contributions

**Christine Strauss:** investigation, writing – original draft, visualization, formal analysis, data curation, methodology. **Daniel Djojic:** investigation, writing – review and editing, visualization, methodology, formal analysis, data curation. **Johannes Stadlmann:** conceptualization, investigation, writing – review and editing, visualization, methodology, formal analysis, data curation. **Josef Georg Grohs:** investigation, methodology, writing – review and editing, supervision, resources. **Jürgen Alphonsus:** investigation, writing – review and editing, methodology, formal analysis, resources. **Sebastian Schmidt:** investigation, writing – review and editing, methodology, resources. **Sabine André:** investigation, methodology, writing – review and editing, resources. **Katharina Margareta Pichler:** investigation, writing – review and editing, methodology, formal analysis. **Sara Savic‐Ivanovic:** investigation, methodology, writing – review and editing, formal analysis, data curation. **Melanie Cezanne:** investigation, methodology, writing – review and editing, formal analysis, project administration, data curation. **Mario Rothbauer:** investigation, methodology, writing – review and editing, formal analysis, supervision. **Reinhard Windhager:** writing – review and editing, supervision, resources, investigation. **Stefan Toegel:** conceptualization, investigation, funding acquisition, writing – original draft, methodology, writing – review and editing, visualization, formal analysis, project administration, supervision.

## Funding

This work was supported by Ludwig Boltzmann Gesellschaft.

## Supporting information


**Data S1:** jsp270194‐sup‐0001‐Supplementary File 1.xlsx.

**Data S2:** jsp270194‐sup‐0002‐supinfo.docx.
**Table S1:**. The mRNA levels of various glycosyltransferases of NP and AF cells as quantified by RT‐qPCR. Numbers indicate relative copy number with respect to SDHA gene expression, which was arbitrarily set to 1000. cDNA from NP and AF cell cultures from 15 donors was pooled and analyzed in duplicate. Functional properties of glycosyltransferases were described in [1].
**Table S2:** LGALS mRNA expression of NP and AF cells using RT‐qPCR. Reference mRNA expression of SDHA was randomly set to 1000. The *p* values of individual cell types were calculated with Friedman's pairwise comparison and comparison of cell types was calculated using the Wilcoxon signed rank test. *n* = 6.
F**igureS1**. Immunocytochemistry staining of AF and NP cell cultivated on glass slide. Gal‐1, Gal‐4, and Gal‐8 with AlexaFluor488 in Zeiss LSM700. Scale bars = 20 μm.

## Data Availability

The data that support the findings of this study are openly available in Zenodo at https://zenodo.org, reference number DOI: 10.5281/zenodo.20020447.
