# Supplementary material for: Galectin‐4: A Novel Mediator of Human Intervertebral Disc Degeneration
Source: JOR Spine. 2026 Aug 6;9(3):e70194. doi: 10.1002/jsp2.70194 (PMC13444353; doi:10.1002/jsp2.70194)
Supplement: Supplementary file 1 [file JSP2-9-e70194-s002.docx]

**Supplementary**

**Table S1**. The mRNA levels of various glycosyltransferases of NP and AF cells as quantified by RT-qPCR. Numbers indicate relative copy number with respect to SDHA gene expression, which was arbitrarily set to 1,000. cDNA from NP and AF cell cultures from 15 donors was pooled and analyzed in duplicate. Functional properties of glycosyltransferases were described in [1].

|  | NP | AF |
| --- | --- | --- |
| N-Glycans | | |
| MAN1 | 63.0 | 119.0 |
| MAN2 | 596.5 | 545.6 |
| MGAT1 | 1.6 | 321.9 |
| MGAT2 | 1,041.7 | 1,046.5 |
| MGAT3 | 1.1 | 1.6 |
| MGAT4A | 5.8 | 5.7 |
| MGAT4B | 1,346.8 | 1,973.8 |
| MGAT5 | 461.9 | 234.0 |
| MGAT5B | 8.2 | 4.7 |
| FUT8 | 485.2 | 505.9 |
| B4GALNT3a | 7.4 | 16.2 |
| ST6Gal1 | 234.3 | 390.1 |
| ST6Gal2 | 28.1 | 26.1 |
| ST3Gal3 | 156.6 | 137.5 |
| ST3Gal4 | 395.0 | 1,055.2 |
| ST3Gal6 | 26.6 | 47.3 |
| O-Glycans | | |
| GALNT | 8,100.4 | 6,226.8 |
| B3GNT | 266.0 | 181.4 |
| GCNT1 | 2,238.5 | 2,913.0 |
| FUT1 | 8.1 | 4.3 |
| 3OST | 14.4 | 10.1 |
| ST3Gal1 | 292.1 | 154.2 |
| ST3Gal2 | 259.5 | 244.7 |
| ST6GalNAc1 | 0.0 | 0.6 |
| ST6GalNAc2 | 1.1 | 30.7 |
| ST6GalNAc3 | 0.3 | 2.0 |
| ST6GalNAc4 | 221.8 | 247.6 |

**Table S2**	LGALS mRNA expression of NP and AF cells using RT-qPCR. Reference mRNA expression of SDHA was randomly set to 1,000. The p-values of individual cell types were calculated with Friedman’s pairwise comparison and comparison of cell types was calculated using the Wilcoxon signed rank test. n = 6

|  | NP | AF | Wilcoxon  Signed rank test |
| --- | --- | --- | --- |
| LGALS1 | 56,519.93 ± 10 920.51 | 53,251.38 ± 7 423.19 | p = 0.345 |
| LGALS2 | 46.76 ± 6.75 | 40.43 ± 5.76 | p = 0.046* |
| LGALS3 | 4,155.21 ± 555.64 | 4,937.88 ± 672.37 | p = 0.028* |
| LGALS4 | 4.21 ± 6.35 | 1.44 ± 0.53 | p = 0.917 |
| LGALS7 | 0.16 ± 0.10 | 0.21 ± 0.17 | p = 0.917 |
| LGALS8 | 312.19 ± 50.96 | 308.60 ± 29.60 | p = 0.753 |
| LGALS9 | 2.58 ± 0.58 | 4.59 ± 2.06 | p = 0.116 |
| **Friedman test** |  |  |  |
| LGALS1 vs LGALS2 | p = 0.016 | |  |
| LGALS1 vs LGALS4 | p < 0.001 | |  |
| LGALS1 vs LGALS7 | p < 0.001 | |  |
| LGALS1 vs LGALS9 | p < 0.001 | |  |
| LGALS2 vs LGALS7 | p = 0.016 | |  |
| LGALS3 vs LGALS4 | p = 0.002 | |  |
| LGALS3 vs LGALS7 | p < 0.001 | |  |
| LGALS3 vs LGALS9 | p = 0.011 | |  |
| LGALS4 vs LGALS8 | p = 0.023 | |  |
| LGALS7 vs LGALS8 | p = 0.001 | |  |

**Fig.S1** Immunocytochemistry staining of AF and NP cell cultivated on glass slide. Gal-1, Gal-4 and Gal-8 with AlexaFluor488 in Zeiss LSM700. Scale bars = 20 µm

**Supplementary File 1.**

N-glycopeptide identification data: The excel file provides both raw and FDR-filtered glyco-PSMs used to generate the glycan-mass histograms in Fig. 5A–B. Raw LC–ESI–MS/MS data were extracted, refined (precursor mass and charge state; no scan merging), and converted to .mgf using PEAKS X Pro Studio 10.6 (build 20,201,221). Spectra were then processed with custom Perl scripts (“SugarQBits”; http://homepage.boku.ac.at/jstadlmann). From PEAKS charge-deconvoluted and deisotoped spectra, only those containing the HexNAc oxonium ion at m/z  204.0867 (±10 ppm) were retained and further evaluated for the co-occurrence (±20 ppm) of three diagnostic signals: the putative Y1 ion [peptide+HexNAc+H]+ and its neutral losses of 203.0794 Da and 120.0423 Da. For spectra containing all three signals, the stored precursor mass (PEPMASS) in the .mgf file was reassigned to the detected Y1 mass (SugarQBits_Kassonade.pl), and common N-glycan oxonium ions (138.0550, 163.0601, 186.0761, 204.0866, 243.0264, 274.0921, 290.0870, 292.1027, 308.0976, 323.2240, 366.1395 Da; ±20 ppm) were removed (SugarQBits_RepX).

Peptide identification was performed with Comet via SearchGUI v4.1.11 (DOI: 10.1021/acs.jproteome.8b00175) against the human reference proteome (UniProt UP000005640) concatenated with reversed decoys, using trypsin with up to one missed cleavage, carbamidomethylation (Cys, fixed), oxidation (Met, variable), and HexNAc (including a 203.0794 Da neutral loss) as a variable modification on Ser/Thr/Asn. Mass tolerances were ±10 ppm for precursors and ±10 ppm for fragments; contaminants were neither included nor excluded. Raw glycopeptide identifications (search rank 1, peptide length > 7, at least one HexNAc-modified residue) are listed in “PEAKS_AFpool_rawIDs” and “PEAKS_NPpool_rawIDs” and were manually filtered by a target–decoy approach to ~1% spectrum-level FDR; the resulting glyco-PSMs are reported in “PEAKS_AFpool_1%fdr_glycoPSMs” and “PEAKS_NPpool_1%fdr_glycoPSMs.” No site localization of N-glycans within peptides was performed. Glycan-mass histograms (Fig. 5A–B) were constructed by spectral counting at a 1 Da bin width from SugarQBits_Kassonade-derived mass differences between the original glycopeptide precursors and the identified peptide sequences for all glyco-PSMs with FDR < 1%; for site-specific histograms (Fig. 5B), only glyco-PSMs (FDR < 1%) covering the respective site were counted.

1.	Toegel, S., et al., *Glycophenotyping of osteoarthritic cartilage and chondrocytes by RT-qPCR, mass spectrometry, histochemistry with plant/human lectins and lectin localization with a glycoprotein.* Arthritis Res Ther, 2013. **15**(5): p. R147.
